# The occurrence of antibiotic-resistant enteric bacteria in Selected Nigerian traditional dairy products

**DOI:** 10.4314/ahs.v22i4.67

**Published:** 2022-12

**Authors:** Roseline E Uzeh, Sylvester Imafidon

**Affiliations:** Department of Microbiology, Faculty of Science, University of Lagos, Lagos, Nigeria

**Keywords:** *Nono*, *wara*, antibiotic resistance, enteric bacteria

## Abstract

**Background:**

*Wara* and *nono* are popular dairy products in Nigeria, rich in nutrients, highly exposed to microbial contaminants during processing and sale and support microbial growth.

**Objectives:**

To investigate occurrence and antibiotic resistance pattern of enteric bacterial pathogens in dairy products.

**Methods:**

Dairy products were serially diluted and cultured on Eosin Methylene Blue agar, *Salmonella-Shigella* agar, McConkey agar and nutrient agar at 37°C for 24 h. Characterisation and identification of isolates with API 20E kit (Biomereux, France). Antibiotic susceptibility was with agar disc diffusion. Polyvalent O and H antisera for *Salmonella* serotyping.

**Results:**

*Salmonella enterica* serovar Enteritidis, *Salmonella enterica* serovar Typhimurium, *Salmonella enterica* serovar Typhi, *Escherichia coli, Klebsiella pneumoniae, Klebsiella oxytoca, Enterobacter aerogenes, Enterobacter cloacae* and *Serratia marcescens*, were identified. Dominant enteric bacterium detected was *E. coli* followed by *Salmonella* spp. Serratia marcescens was the least occurring. The isolates were most resistant to trimethoprim-sulfamethoxazole (96.7%), amoxicillin (83.3%), augmentin (83.3%), chloramphenicol (66.7%), streptomycin (50%). They were resistant to ≥ 4 (multiple) antibiotics, *E. coli* 8, *Salmonella* spp. 7, *Serratia marcescens* 6 and *Klebsiella* spp. and *Enterobacter* spp. 4 each.

**Conclusion:**

The presence of enteric bacterial pathogens in *wara* and *nono* and their resistance to multiple antibiotics was reported in this study.

## Introduction

Dairy foods are made from milk, they are highly nutritious and therefore of great value in the human diet. Most African traditional dairy foods are however exposed to contamination right from when the milk is collected, all through processing and up to the post-processing stage, during retail. *Wara* and *nono* are widely consumed dairy products in Nigeria, although they are most common in the Northern part of the country among the Fulanis. *Wara* is an unripened cheese made by coagulating milk with the leaf extract of Sodom Apple (*Calotropis procera*). It is cut and reintroduced into whey while on retail. On the other hand, *nono* is soured milk, like yoghurt, it is produced by inoculating freshly collected milk in calabash with a little quantity of previously fermented *nono* as starter culture or by exposing the milk to chance inoculation by microorganisms majorly from the environment and allowing it to ferment for about 24 hours. *Wara* and *nono* are made at the cottage level for domestic and commercial purpose. They serve as a rich source of nutrients for the growth of microorganisms that come in contact with them^4^. *Nono* and *wara* are likely to be contaminated by enteric organisms from animals through the unpasteurised milk used for their production and the handlers during processing and even post-processing during retail. As a result of poor hygiene and crude milking process, the dairy products are generally prone to microbial contamination^1^, which pose great health risks such as colitis, diarrhoea, dysentery and other severe enteric diseases^15^. This has led to morbidity among many consumers^6^.

Enteric bacteria are found in the gut of animals including humans either as commensals, opportunistic pathogens or pathogens. They can get transferred to food through faecal matter from animals and humans especially where strict hygiene has been compromised by food handlers. They are in the family, Enterobacteriaceae among which are the genera *Salmonella, Citrobacter, Escherichia, Enterobacter*, *Serratia* and *Klebsiella*. Enteric bacterial pathogens have been reported to be characterized by a high level of resistance to antibiotics^9^;^3^, which further intensifies gastro-intestinal morbidity with high resultant illnesses. The foodborne illness from consumption of *wara* and *nono* may not have been well documented.

This study was aimed to isolate and identify enteric bacteria from retail wara and nono and determine the antibiotic resistance profile of the enteric bacteria and in all, evaluate the safety of the two traditional dairy products.

## Materials and Methods

### Sample collection

*Wara* and *nono* were purchased randomly from retailers in Lagos. Wara samples (60) were obtained from markets in Ketu, Ikorodu, Mile 12, Oshodi, Ojota and Bariga, while nono (60 samples) were purchased from hawkers in Kara, Agege, Yaba, Ojodu, Oworo and Ogba all in Lagos metropolis. *Wara* samples were collected into sterile containers and *nono* samples were purchased as already packaged in retailing bottles and transported in the cold chain to the Microbiology laboratory, the University of Lagos for analyses.

### Determination of pH of wara and nono samples

The pH level of each sample was determined in a 20 ml homogenized suspension.

### Isolation of enteric bacteria from *wara* and *nono*

Ten grams (10 g) of *wara* was weighed and homogenized in 90 ml of normal saline in a conical flask. The suspension was filtered and the filtrate was used for 10-fold serial dilutions in Tryptic Soy broth and incubated for 24 h at 37°C. From the dilutions, 1 ml was plated on Eosin Methylene Blue (EMB) agar, *Salmonella-Shigella* (SS) agar, MacConkey agar, nutrient agar and incubated for 24 h at 37°C. The same method was used for nono except that 10ml of *nono* was homogenized in 90 ml of normal saline. Colonies from primary culture plates were subcultured to obtain pure colonies which were subsequently inoculated on nutrient agar slants and incubated for 24 h at 37°C and after growth it was stored in the refrigerator at 4°C.

### Identification of isolated enteric bacterial isolates from *wara* and *nono*

Pure colonies were Gram stained. Characterisation and identification of isolates were carried out using the API 20 E kit (Biomereux, France). A suspension of the bacterial colony was made in sterile distilled water. Each of the 20 separate compartments of the API test strip was filled with bacterial suspension. Sterile oil was added to the compartments for decarboxylation of arginine, lysine, ornithine, production of hydrogen sulphide and test for urease. Some drops of water were added to the tray and the API test strip was put on the tray, covered, labelled and incubated at 37°C for 24 h. At the end of incubation, strips were observed for colour change and results recorded. A positive result was revealed by the change of the bromocresol purple indicator contained in the API 20 E medium to yellow. The biochemical profiles obtained for the isolates were read using the identification tables and web software.

### Serotyping of *Salmonella* isolates

Salmonella strains were typed with polyvalent O and H antisera by using a slide agglutination test according to Collins and Lyne^8^. One drop of sterile normal saline was placed at both ends of a clean glass slide. A pure colony was added to each and emulsified to form a smooth suspension. A drop of the antiserum was added to one of the suspensions, mixed by rocking the slide to and fro for 30 – 60 seconds and examined for the presence of agglutination. The second suspension without antiserum served as a control.

### Antibiotic susceptibility test

The antibiotic susceptibility testing was done using the agar disc diffusion technique. Pure isolates were inoculated into tubes containing tryptic soy broth and incubated at 37°C for 24 h. The turbidity of the broth culture was standardized to match that of the 0.5 McFarland standard, which corresponds approximately to 1.5 × 108 CFU/ml. Each isolate was investigated for antibiotic susceptibility with the disc diffusion method by spreading 0.5 McFarland turbid pure strain on Mueller-Hinton agar plates with a sterile cotton swab, and the antibiotic discs were placed on it with the help of sterile forceps. The discs were pressed gently to ensure adequate contact with the inoculated agar. Antibiotics tested were ofloxacin (5 µg), ciprofloxacin (5 µg), trimethoprim-sulfamethoxazole (25 µg), amoxicillin (30 µg), chloramphenicol (30 µg), sparfloxacin (10 µg), augmentin (30 µg), gentamicin (10 µg), pefloxacin (30 µg), and streptomycin (10 µg). After incubation at 37°C for 24 h, the zones of inhibition were measured using callipers, recorded and interpreted using recommended standards (CLSI^7^).

### Data analysis

Data was analysed for descriptive statistics using 2016 version of Microsoft Excel software and results were expressed in percentages. The two-tailed student t-test was used to compare the difference in the bacterial population of the two dairy products with significance at p <0.05

## Result

**Figure 1:**
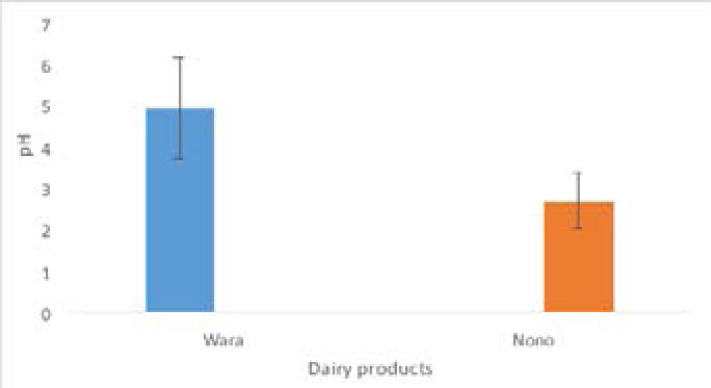
Mean pH values of *wara* and *nono*

**Figure 2:**
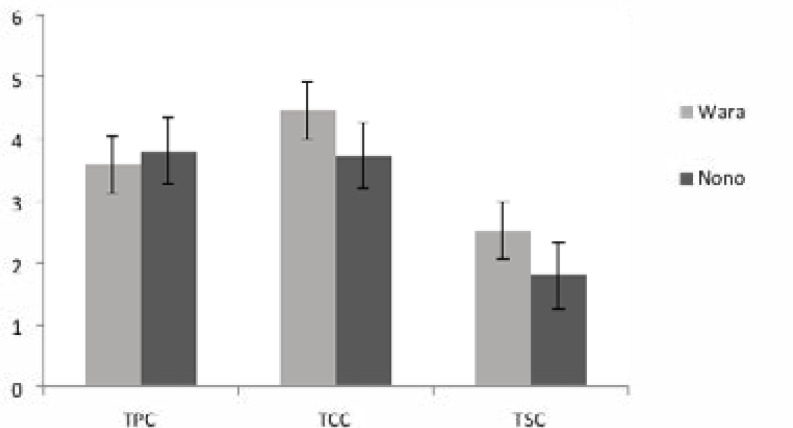
Bacterial enumeration in *wara* and *nono* (TPC- Total Plate Count (× **10^8^ CFU/g**); TCC- Total Coliform Count (×**10^6^ CFU/g**); TSC- Total Salmonella Count (×**10^5^ CFU/g**)

pH and occurrence of enteric bacteria in wara and nono The mean pH values of wara and nono were 4.93 and 2.68 respectively (Figure 1), while the mean total plate count was 3.59 x 108 CFU/g for wara and 3.81 x 108 CFU/ml for nono. The mean coliforms and Salmonella count was higher in wara than nono with a coliform count of 3.73 x 106 CFU/g for nono and 4.47 x 106 CFU/ml for wara, while for Salmonella count it was 1.80 x 105 CFU/g and 2.52 x 105 CFU/ml for nono and wara respectively (Figure 2). However, the difference in the bacterial count of the two dairy products was not significant at p >0.05.

A total of 30 isolates of members of Enterobacteriaceae were isolated from the two dairy products with 17 isolates from *wara* and 13 from *nono*. The isolates were identified as *Salmonella enterica* serovar Enteritidis, *Salmonella enterica* serovar Typhimurium, *Salmonella enterica* serovar Typhi, *Escherichia coli, Klebsiella pneumoniae, Klebsiella oxytoca*, En*terobacter aerogenes, Enterobacter cloacae*, and *Serratia marcescens*. Among all the isolates, *E. coli* 12 (40%) was predominant followed by *Salmonella* spp. 9 (30%) and the least occurring was *Serratia marcescens* 2 (6.7%) (Table 1).

**Table 1:** Frequency of occurrence of isolated enteric bacteria in *wara* and *nono*

Isolated bacteria	*Wara*	*Nono*	Total frequency of occurrence (%)
*Salmonella* spp.	6	3	9 (30)
*Escherichia coli*	6	6	12 (40)
*Klebsiella* spp.	3	1	4 (13.3)
*Enterobacter* spp.	1	2	3 (10)
*Serratia marcescens*	1	1	2 (6.7)
Total	17	13	30 (100)

### Prevalence of antibiotic resistance among enteric bacteria isolated from *wara* and *nono*

The antibiotic susceptibility pattern of enteric bacteria isolated from *wara* and *nono* were relatively similar, all isolates were susceptible to ofloxacin (Table 2). The antibiotic resistance profile of the isolates is as shown in Table 3. All the isolates were resistant to ≥ 4 (multiple) antibiotics. Strains of *E. coli* were resistant to 8(80%) antibiotics, *Salmonella* spp to 7 (70%), *Serratia marcescens* to 6 (60%), *Klebsiella* spp. and *Enterobacter* spp. each to 4 (40%) antibiotics. Generally, from the results of the antibiotic resistance, the highest number of isolates were resistant to trimethoprim-sulfamethoxazole 29 (96.7%) followed by augmentin and amoxicillin 25 (83.3%) to each, 20 (66.7%) to chloramphenicol and 15 (50%) to streptomycin (Table 3). Some strains were completely (100%) resistant to some of the antibiotics as shown in Table 4.

**Table 2:** Antibiotic susceptibility pattern of enteric bacteria isolated from *wara* and nono

Isolate code (distribution)	Zones of inhibition (mm)									

	SPX	CPX	AMX	AUG	GEN	PEF	OFX	STR	TIM	CHL
***Salmonella*** spp.										
G17^W^	S(26)	S(28)	S(17)	R(0)	R(0)	S(28)	S(29)	S(21)	R(0)	S(23)
I8^W^	S(22)	S(21)	R(13)	R(0)	S(19)	S(26)	S(24)	S(17)	I(14)	I(14)
L18^W^	S(20)	S(26)	S(16)	R(11)	S(15)	S(26)	S(28)	S(16)	R(0)	R(11)
M20^N^	S(20)	S(27)	R(0)	R(0)	S(19)	S(21)	S(27)	S(18)	I(14)	I(13)
M8^N^	S(21)	S(25)	S(16)	R(0)	S(16)	R(17)	S(29)	S(15)	I(14)	I(13)
I14^W^	S(21)	S(28)	R(0)	R(0)	S(16)	S(24)	S(21)	S(16)	R(0)	S(18)
WJ1^W^	S(22)	S(25)	S(17)	S(14)	S(16)	S(26)	S(29)	S(17)	R(0)	I(16)
P20^N^	S(19)	S(27)	R(0)	S(14)	S(16)	S(26)	S(29)	S(17)	R(0)	I(13)
J13^W^	S(20)	S(30)	R(0)	R(0)	S(16)	S(22)	S(21)	I(14)	R(0)	I(17)
***Escherichia coli***										
I2^W^	S(24)	S(26)	R(0)	R(0)	S(20)	S(27)	S(28)	R(0)	R(0)	S(20)
J20^W^	S(25)	S(27)	R(0)	R(0)	S(19)	S(26)	S(29)	R(0)	R(0)	I(17)
M5^N^	S(19)	S(23)	R(0)	R(0)	S(21)	S(19)	S(21)	R(0)	R(0)	R(0)
H6^W^	S(22)	S(25)	R(0)	R(0)	S(19)	S(27)	S(29)	R(0)	R(0)	S(21)
N13^N^	R(12)	S(25)	R(0)	R(0)	S(20)	R(13)	S(17)	R(0)	R(0)	I(16)
M4^N^	S(24)	S(28)	R(0)	R(0)	S(17)	S(26)	S(30)	R(0)	R(0)	S(21)
Q9^N^	R(12)	I(17)	R(0)	R(0)	S(17)	S(26)	S(30)	R(0)	R(0)	R(11)
H14^W^	R(14)	I(20)	R(0)	R(0)	S(21)	R(12)	S(19)	R(0)	R(0)	I(15)
P16^N^	S(25)	S(33)	R(0)	R(0)	S(19)	S(26)	S(30)	R(0)	R(0)	S(22)
N6^N^	S(26)	S(35)	R(0)	R(0)	S(18)	S(31)	S(27)	R(0)	R(0)	S(22)
G14^W^	S(28)	S(33)	R(0)	R(0)	S(18)	S(29)	S(25)	R(0)	R(0)	S(21)
L12^W^	S(25)	S(30)	R(0)	R(0)	S(19)	S(26)	S(29)	R(0)	R(0)	S(20)
***Klebsiella*** spp.										
K19^W^	S(24)	S(27)	R(0)	S(14)	S(16)	S(23)	S(23)	S(15)	R(0)	R(12)
H3^W^	S(27)	S(27)	R(0)	S(14)	S(16)	S(25)	S(26)	S(15)	R(0)	R(12)
R9^N^	S(23)	S(27)	R(0)	R(0)	S(15)	S(24)	S(26)	S(17)	I(11)	R(11)
L10^W^	S(26)	S(24)	R(0)	S(14)	S(16)	S(25)	S(24)	S(17)	R(0)	I(14)
***Enterobacter*** spp.										
Q19^N^	S(24)	S(29)	R(0)	R(0)	S(16)	S(24)	S(27)	S(20)	I(11)	I(14)
R16^N^	S(27)	S(26)	R(0)	R(0)	S(17)	S(26)	S(25)	S(18)	I(11)	I(15)
H2^W^	S(26)	S(23)	R(0)	R(0)	S(19)	S(24)	S(29)	S(19)	I(11)	S(18)
***Serratia*** spp.										
G12^W^	S(26)	S(24)	S(17)	R(0)	S(16)	S(26)	S(29)	R(11)	R(0)	R(0)
N4^N^	S(26)	S(28)	R(13)	R(0)	R(11)	S(26)	S(27)	I(12)	S(16)	R(0)

S: Susceptible; I: Intermediate; R: Resistant; SPX: Sparfloxacin (10 µg), CPX: Ciprofloxacin (5 µg), AMX: Amoxicillin (30 µg), AUG: Augmentin (30 µg), GEN: Gentamicin (10 µg), PEF: Pefloxacin: (5 µg), OFX: Ofloxacin: (5 µg), STR: Streptomycin (10 µg), TIM: Trimethoprim-sulfamethoxazole (25 µg), CHL: Chloramphenicol (30 µg). Isolates from wara: W, isolates from nono: ^N^

**Table 3:** Antibiotic resistance of enteric bacteria isolated from *wara* and *nono*

Enteric bacteria	Total isolates	Number of resistant isolates (%)									
		SPX	CPX	AMX	AUG	GEN	PEF	OFX	STR	TIM	CHL
*Escherichia coli*	12	3(25)	2(16.7)	12(100)	12(100)	0(0)	2(16.7)	0(0)	12(100)	12(100)	5(41.7)
*Salmonella* spp.	9	0(0)	0(0)	5(55.6)	7(78.2)	1(11.1)	1(11.1)	0(0)	1(11.1)	9(100)	7(77.8)
*Klebsiella* spp.	4	0(0)	0(0)	4(100)	1(25)	0(0)	0(0)	0(0)	0(0)	4(100)	4(100)
*Enterobacter* spp.	3	0(0)	0(0)	3(100)	3(100)	0(0)	0(0)	0(0)	0(0)	3(100)	2(66.7)
*Serratia marcescens*	2	0(0)	0(0)	1(50)	2(100)	1(50)	0(0)	0(0)	2(100)	1(50)	2(100)
Total	30	3(10)	2(6.7)	25(83.3)	25(83.3)	2(6.7)	3(10)	0(0)	15(50)	29(96.7)	20(66.7)

• Isolates that showed intermedia te resistance were also classified as resistant

SPX: Sparfloxacin (10 µg), CPX: Ciprofloxacin (5 µg), AMX: Amoxicillin (30 µg), AUG: Augmentin (30 µg), GEN: Gentamicin (10 µg), PEF: Pefloxacin: (5 µg), OFX: Ofloxacin: (5 µg), STR: Streptomycin (10 µg), TIM: Trimethoprim-sulfamethoxazole (25 µg), CHL: Chloramphenicol (30 µg).

**Table 4:** Enteric bacterial isolates from dairy products with 100% resistance to antibiotics

Enteric bacteria	Antibiotics
*Escherichia coli*	streptomycin, augmentin, trimethoprim- sulfamethoxazole and amoxicillin
*Salmonella* spp.	trimethoprim-sulfamethoxazole
*Serratia marcescens*	chloramphenicol, augmentin and streptomycin
*Klebsiella* spp.	chloramphenicol, amoxicillin and trimethoprim- sulfamethoxazole
*Enterobacter* spp.	trimethoprim-sulfamethoxazole, amoxicillin and augmentin

## Discussion

Among the commonly retailed traditional dairy products in Lagos, *nono* and *wara* are considered as major products consumed by the populace but a high level of contamination by enteric bacterial pathogens which pose a health risk to the public was obtained in this study. The enteric bacteria isolated included *Salmonella enterica*, serovar Enteritidis, *Salmonella enterica* serovar Typhimurium, *Salmonella enterica* serovar Typhi, *Escherichia coli, Klebsiella pneumoniae*, *Klebsiella oxytoca, Enterobacter aerogenes, Enterobacter cloacae* and *Serratia marcescens*. Similar resultwere obtained by previous workers^12^;^1^. In this study, *Escherichia coli*, a faecal coliform, was predominant 12(40%) which is an indication of the presence of enteric pathogens in the dairy products, this was established in our result where *E. coli* was followed by *Salmonella* spp. 9(30%) in terms of abundance. This result is similar to that of Yilma et al.^16^ who in their dairy products in Egypt found a correlation in the abundance of faecal coliform and enteric pathogens. The detection of Salmonella from this study is, however, in contrast to the result obtained by Sobeih et al.^13^, they did not detect *Salmonella* from the raw milk and dairy products they investigated. The enteric pathogens from our study may have been from the handlers, the animals from which the milk for the dairy products was obtained, the dairy farm environment and the processing water. The processing of the dairy products often takes place in the dairy farms because, immediately after the farmers milk their cows, the excess milk is processed into varieties of products among which are *nono* and *wara*. The farms are usually laden with faeces from cows which may contaminate utensils, water and equipment in the farm that are used for processing the dairy products. There may also be a high level of cross-contamination of the final products with the contaminated soil, utensils, and handlers. The enteric pathogens may also be directly from the handlers if they fail to maintain strict personal hygiene. The source of water is surface water which is exposed to contamination by enteric bacteria from dwellers in the locality that visit the stream for various activities and also as a result of runoffs into such water bodies. Poor packaging and exposure of both wara and nono, while they are displayed for sale in bowls, can also serve as a source of contamination as previously reported^14^;^2^;^11^.

In this study, the result of the antibiotic susceptibility test showed that most of the enteric bacterial isolates were resistant to multiple antibiotics, which included chloramphenicol, amoxicillin, trimethoprim-sulfamethoxazole, gentamicin, ciprofloxacin, streptomycin and augmentin. This is similar to the result of Chauhan *et al.*^5^ who isolated multidrug-resistant *Klebsiella pneumoniae from raw milk. In the same vein, Fakruddin et al.^10^ demonstrated the occurrence of multidrug-resistant Enterobacter spp., Klebsiella spp. and Citrobacter spp. in powder milk and other food samples. The antibiotic-resistant enteric pathogens present in the dairy foods will further increase antibiotic pressure where resistant strains will dominate the intestinal microbiome which will cause limited effective treatment of infections caused by the enteric pathogens*

## Conclusion

The Nigerian dairy products; *nono* and *wara*, were found to be contaminated with enteric pathogens which were resistant to multiple antibiotics. The results of this investigation will contribute to the collection of data on the safety status of these traditional dairy products which will be very useful in the protection of the health of the consumers. There is a need for regular surveillance and routine examination of these products.
